# ML345 is a potent and selective NLRP3 inflammasome inhibitor with anti-inflammatory activity

**DOI:** 10.1186/s43556-025-00363-7

**Published:** 2025-11-13

**Authors:** Hualong Lin, Xinxin Liang, Weijie Hao, Xiaoli Lu, Bo Li, Xiaohong Wang

**Affiliations:** 1 Department of Gynecology and Obstetrics, Tangdu Hospital, Fourth Military Medical University, Xi’an, Shaanxi Province China; 2 Xi’an International University, Yanta District, 18 Yudou Road, Xi’an, Shaanxi 710077 People’s Republic of China

**Keywords:** ML345, NLRP3 inflammasome, NLRP3 inhibitor, NEK7, Inflammatory diseases, Miscarriage

## Abstract

Excessive activation of the NOD-like receptor pyrin domain–containing protein 3 (NLRP3) inflammasome plays a key role in the pathogenesis of various inflammatory diseases. Despite the development of several NLRP3 inhibitors, no specific therapy has been approved for clinical use, underscoring the urgent need for safe and effective agents. Here, we demonstrate that ML345 acts as a highly potent and selective NLRP3 inhibitor with strong therapeutic potential for NLRP3-driven inflammation. ML345 effectively suppresses canonical, noncanonical, and alternative NLRP3 inflammasome activation pathways, without affecting other inflammasomes. Mechanistically, ML345 blocks NLRP3 inflammasome activation independently of its intrinsic insulin-degrading enzyme (IDE) inhibitory activity. ML345 binds to NLRP3 in a non-covalent manner and directly targets tyrosine 381 (Y381), disrupting its essential interaction with NIMA-related kinase 7 (NEK7), consequently preventing inflammasome complex formation. In vivo, ML345 is well tolerated and markedly alleviates inflammatory responses and pathology in mouse models of NLRP3-associated disorders, including systemic inflammation and miscarriage triggered by lipopolysaccharide (LPS). Compared with several previously reported NLRP3 inhibitors, ML345 exhibits superior selectivity and comparable or greater inhibitory potency. These findings establish ML345 as a safe and selective NLRP3 inhibitor with robust anti-inflammasome effects and highlight its potential as a promising therapeutic candidate for NLRP3-driven diseases.

**Supplementary Information:**

The online version contains supplementary material available at 10.1186/s43556-025-00363-7.

## Introduction

NLRP3 is a key cytosolic sensor that recognizes a wide range of danger signals, encompassing pathogen-associated molecular patterns (PAMPs) such as nigericin, viral RNA, and pore-forming toxins, as well as damage-associated molecular patterns (DAMPs) including cholesterol or uric acid crystals, extracellular ATP, and aggregated proteins [1, 2]. Upon activation by these stimuli, NLRP3 interacts with NEK7 and recruits ASC, an apoptosis-associated speck-like protein containing a CARD, which subsequently engages the cysteine protease pro-caspase-1, leading to the formation of the inflammasome complex [3–5]. Caspase-1 is then activated, driving the processing of IL-1β and IL-18 and inducing pyroptosis via gasdermin D cleavage, which is critical for pathogen defense and immune homeostasis [6, 7]. However, excessive triggering of the NLRP3 inflammasome can directly contribute to multiple inflammatory diseases, including sepsis, atherosclerosis, Parkinson’s disease, type 2 diabetes, multiple sclerosis, and cryopyrin-associated periodic syndromes (CAPS) [8–12]. Thus, pharmacological modulation of the NLRP3 inflammasome represents a promising strategy for mitigating inflammation-related disorders.

Several IL-1β–blocking biologics, such as anakinra, canakinumab, and rilonacept, have received clinical approval for treating NLRP3-associated diseases [13]. These agents have demonstrated therapeutic efficacy in CAPS linked to NLRP3 hyperactivation and have also been evaluated in other inflammasome-driven disorders. However, activation of the NLRP3 inflammasome triggers not only IL-1β release but also pyroptosis and the secretion of additional proinflammatory cytokines, such as IL-18, all of which contribute to disease pathogenesis [14–16]. Moreover, IL-1β production can result from activation of inflammasomes other than NLRP3 or via inflammasome-independent pathways, raising concerns that IL-1β-specific blockade may impair host defense and increase infection risk [17–19]. Therefore, direct suppression of the NLRP3 inflammasome could provide an improved and well-tolerated therapeutic option for NLRP3-driven diseases.

Recent studies have identified several small molecules that inhibit NLRP3 inflammasome activation and demonstrate therapeutic potential in preclinical models, including MCC950, oridonin, CY-09, OLT1177, tranilast, RRx-001, and entrectinib [11, 20–22]. However, no NLRP3-targeting inhibitor has yet received clinical approval. Among these, oridonin, CY-09, RRx-001, and entrectinib remain in the preclinical stage and have not advanced to clinical trials. The MCC950 derivatives inzomelid (NCT04015076) and IZD334 (NCT04086602) have recently finished phase I studies, while tranilast (NCT03923140) and OLT1177 (NCT05658575) are under phase II evaluation for CAPS and acute gouty arthritis, respectively [11, 23, 24]. Although several NLRP3 inhibitors have already reached the stage of clinical evaluation, their clinical application is still distant. Developing effective, specific, and safe NLRP3 inflammasome inhibitors remains crucial.

ML345 was identified through an ultra-high-throughput screening (uHTS) campaign and has been characterized as a potent small-molecule inhibitor of insulin-degrading enzyme (IDE) [25]. It exerts its inhibitory effect by targeting the Cys819 residue within IDE and exhibits favorable cell permeability and chemical stability. The inhibition is reversible and can be attenuated by reducing agents such as β-mercaptoethanol or dithiothreitol [25]. As IDE is the principal protease responsible for insulin degradation in vivo, ML345 and its derivatives are considered to enhance insulin signaling and hold promise for the treatment of diabetes. However, pharmacological actions and molecular targets of ML345 in inflammation have yet to be elucidated.

In this study, we demonstrate that ML345 is a selective and potent inhibitor of the NLRP3 inflammasome with a favorable safety profile. Mechanistically, ML345 binds directly to the Y381 residue of NLRP3, thereby disrupting its interaction with NEK7, a critical step in inflammasome assembly and activation. Furthermore, ML345 demonstrated robust in vivo efficacy while being well tolerated, mitigating diverse NLRP3-driven pathologies, including systemic inflammation and miscarriage triggered by LPS. Importantly, ML345 exhibited selectivity and inhibitory potency comparable or superior to several previously reported NLRP3 inhibitors. Taken together, our results highlight ML345 as a promising lead compound for developing NLRP3-targeted therapies against inflammatory diseases.

## Results

### ML345 is a novel, broad-spectrum inhibitor of the NLRP3 inflammasome

**Fig. 1:**
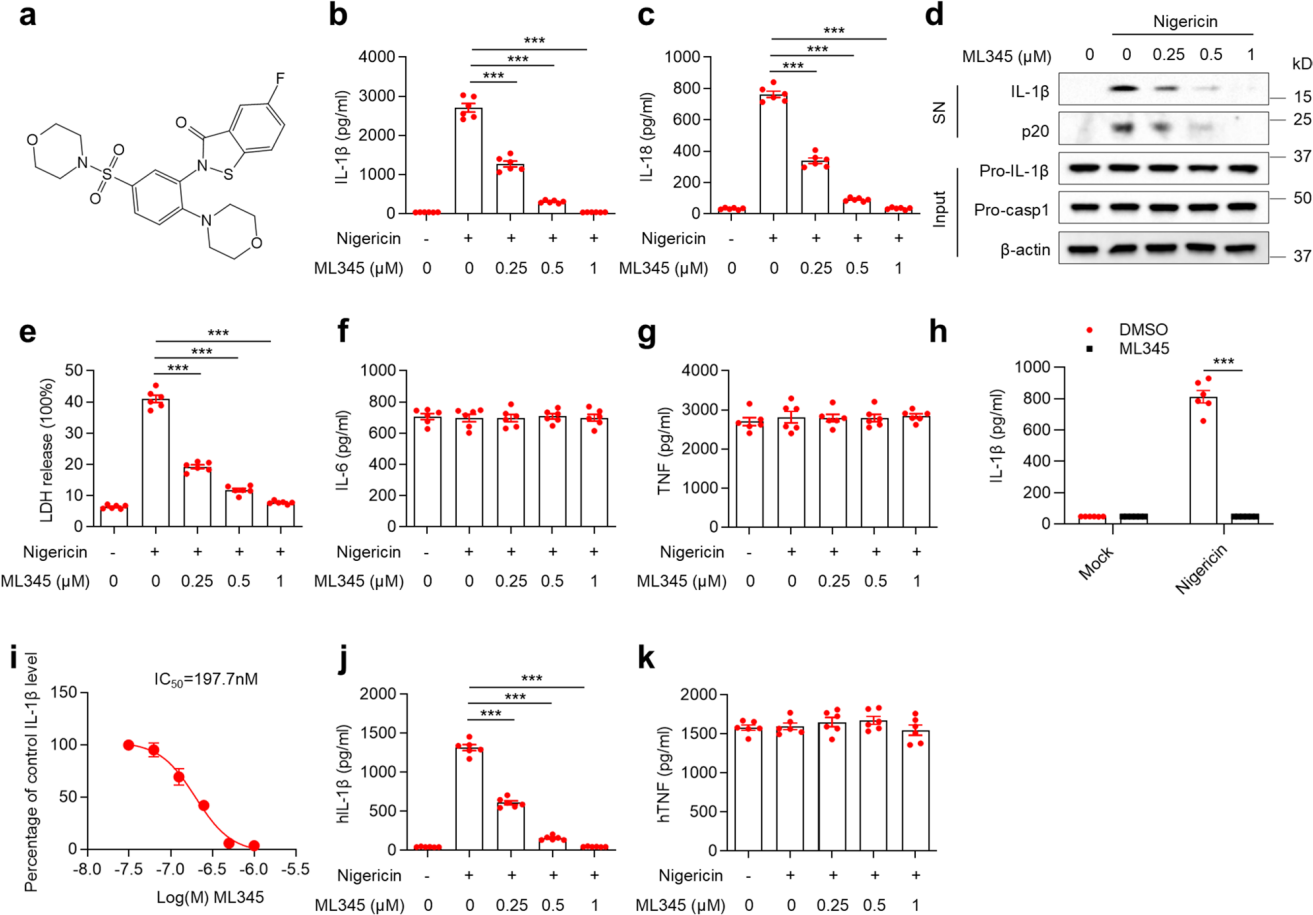
ML345 inhibits nigericin-induced inflammatory cytokine secretion and pyroptosis. **a–i** BMDMs or iBMDMs were treated with ML345 and then stimulated with nigericin. **a** Structure of ML345. **b**,** c** Levels of IL-1β and IL-18 in supernatants (SN) were measured by ELISA. **d** Immunoblot showing secreted IL-1β and cleaved caspase-1 (p20) in SN, and pro-IL-1β/pro-casp1 in cell lysates (input). **e** LDH release in SN. **f–i** IL-6, TNF, and IL-1β secretion measured by ELISA in BMDMs (**f**,** g**,** i**) or iBMDMs (**h**). **j** and **k** Human PBMCs were treated with ML345 and then stimulated with nigericin. IL-1β and TNF levels in SN were measured by ELISA. Data are mean ± SEM from six (**b, c**,** e–h, j**,**k**) or three (**i**) independent experiments. Statistical significance was assessed using one-way ANOVA (**b**,** c**,** e**,** j**) or two-way ANOVA (**h**). ***p < 0.001

The effect of ML345 on NLRP3 inflammasome activation was assessed in LPS-primed primary bone marrow–derived macrophages (BMDMs) following stimulation with the canonical activator nigericin. ML345 dose-dependently suppressed IL-1β and IL-18 secretion, as well as caspase-1 cleavage (Fig. 1a–d). In line with the role of NLRP3 in pyroptosis, ML345 also reduced nigericin-induced lactate dehydrogenase (LDH) release, while IL-6 and TNF secretion remained unaffected (Fig. 1e–g). Consistently, in immortalized BMDMs (iBMDMs), ML345 markedly decreased IL-1β release (Fig. 1h). Quantitative analysis revealed an IC₅₀ of approximately 197.7 nM for ML345-mediated inhibition of IL-1β release (Fig. 1i). These findings suggest that ML345 potently blocks inflammasome-driven cytokine maturation and pyroptosis.

In primary human PBMCs stimulated with nigericin, ML345 dose-dependently reduced IL-1β secretion without altering TNF production (Fig. 1j-k), demonstrating its inhibitory activity across species.

**Fig. 2:**
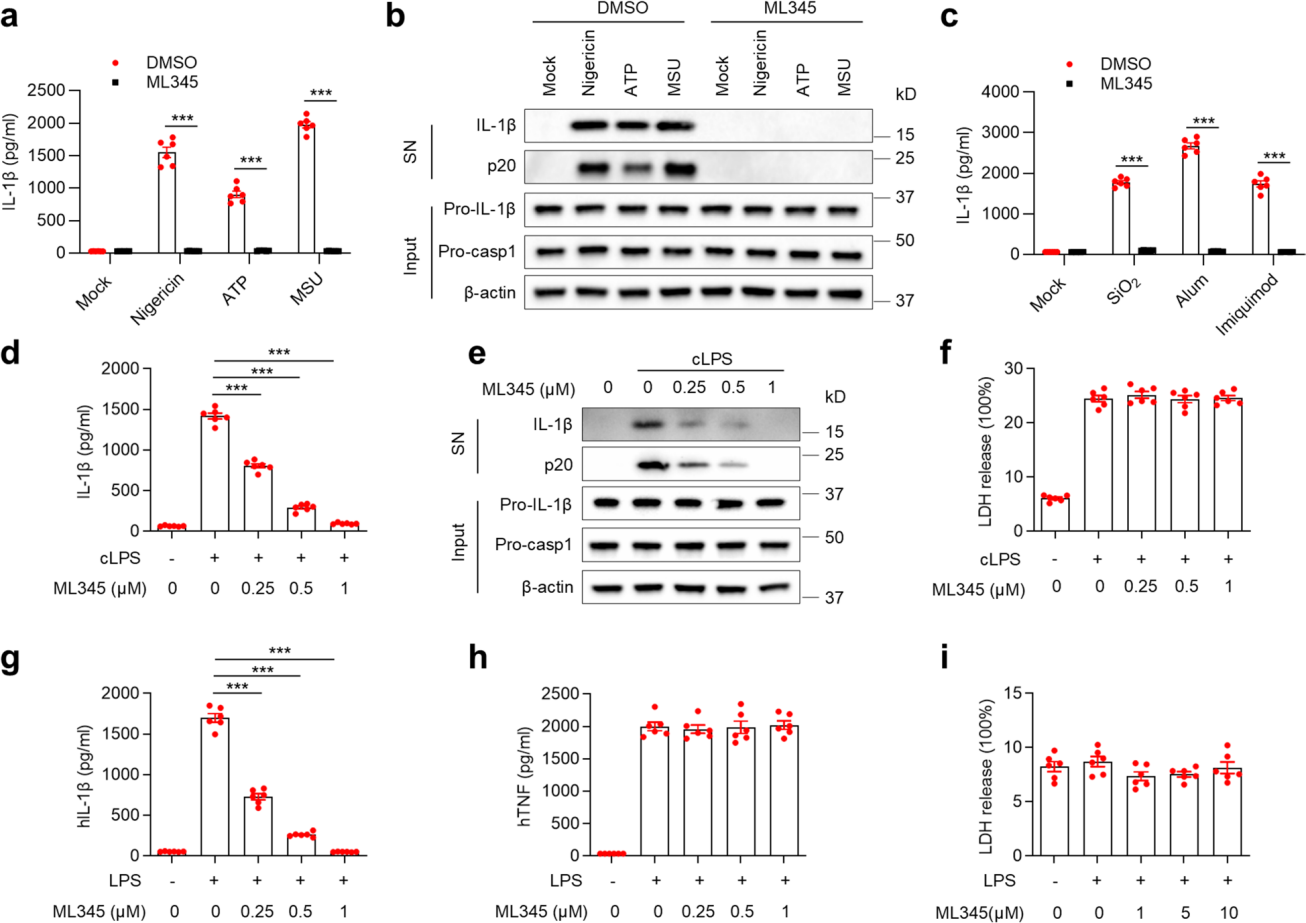
ML345 broadly suppresses NLRP3 inflammasome activation. **a–c** BMDMs were treated with ML345 and then stimulated with nigericin, ATP, or MSU, or with particulate (SiO₂, alum) and small-molecule (imiquimod) activators. **a**,** c** IL-1β levels in SN were quantified by ELISA. **b** Immunoblot showing secreted IL-1β and p20 in SN, and pro-IL-1β/pro-casp1 in input. **d–f** BMDMs were treated with ML345 before LPS transfection.** d** IL-1β secretion measured by ELISA. **e** Immunoblot as described above. **f** LDH release in SN. **g** and **h** Human PBMCs were treated with ML345 and stimulated with LPS. IL-1β and TNF levels in SN were measured by ELISA. **i** BMDMs were treated with ML345 before LPS stimulation. LDH release in SN was quantified. Data are mean ± SEM from six independent experiments. Statistical significance was assessed using one-way ANOVA (**d**,** g**) or two-way ANOVA (**a**,** c**). ***p < 0.001

Apart from nigericin, canonical NLRP3 inflammasome activation can occur in response to ATP, MSU, silica (SiO₂), alum, and imiquimod [26–28]. ML345 effectively inhibited IL-1β secretion and caspase-1 cleavage upon stimulation with these agents (Fig. 2a–c). Furthermore, intracellular LPS (cLPS) activates the noncanonical caspase-11 pathway, resulting in NLRP3-dependent IL-1β release [29–31]. ML345 attenuated IL-1β release and caspase-1 processing after cLPS stimulation, while leaving LDH release unchanged (Fig. 2d–f). In human PBMCs, LPS alone can activate the alternative NLRP3 inflammasome [32]. ML345 significantly reduced IL-1β secretion without affecting TNF production (Fig. 2g-h). Importantly, ML345 exhibited no detectable cytotoxicity, even at elevated concentrations (Fig. 2i). Collectively, these findings establish ML345 as a novel, broad-spectrum, and well-tolerated NLRP3 inflammasome inhibitor.

### ML345 selectively inhibits NLRP3 inflammasome activation without affecting other inflammasomes

**Fig. 3:**
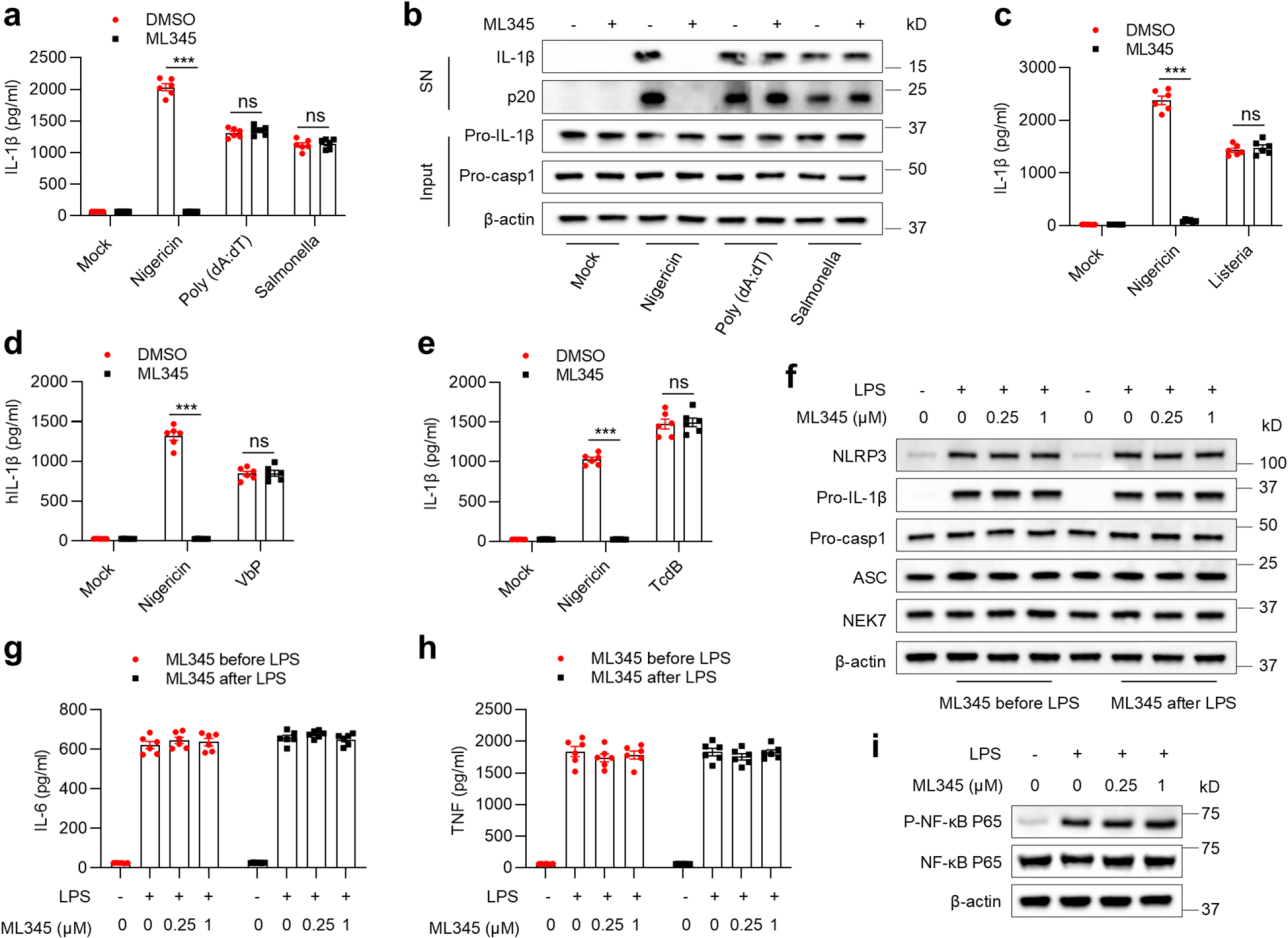
ML345 specifically inhibits NLRP3 inflammasome activation. **a–c** BMDMs were treated with ML345 and then stimulated with nigericin, poly(dA:dT), *Salmonella*, or *L. monocytogenes* (Listeria)*.* **a**,** c** IL-1β levels in SN were measured by ELISA. **b** Immunoblot showing secreted IL-1β and p20 in SN, and pro-IL-1β/pro-casp1 in input. **d** and** e** THP-1 cells (**d**) or BMDMs (**e**) were treated with ML345 before stimulation with nigericin, VbP, or TcdB. IL-1β secretion was measured by ELISA. **f–h** BMDMs were treated with ML345 pre- or post-LPS stimulation. (**f**) Immunoblot showing NLRP3, pro-IL-1β, pro-casp1, ASC and NEK7 in input. **g**,** h** IL-6 and TNF secretion measured by ELISA. (**i**) Immunoblot of phosphorylated and total NF-κB p65 in BMDMs treated with ML345 before LPS stimulation. Data are mean ± SEM from six independent experiments. Statistical significance was assessed using two-way ANOVA (**a**,** c–e**). ***p < 0.001, ns, not significant

Beyond NLRP3, several other inflammasomes drive IL-1β secretion [18]. To assess the specificity of ML345, BMDMs were exposed to poly(dA:dT) or infected with *Salmonella enterica serovar* Typhimurium, which activate the AIM2 and NLRC4 inflammasomes, respectively. Unlike its strong inhibition of NLRP3 activation, ML345 had no effect on IL-1β secretion or caspase-1 cleavage induced by AIM2 or NLRC4 (Fig. 3a-b). Furthermore, accumulating evidence suggests that *Listeria monocytogenes*, Val-boroPro (VbP), and *Clostridium difficile* toxin B (TcdB) activate the NLRP6, NLRP1/CARD8, and Pyrin inflammasomes, respectively. ML345 likewise failed to block IL-1β secretion mediated by NLRP6, NLRP1/CARD8, or Pyrin inflammasomes (Fig. 3c–e). Collectively, the data indicate that ML345 is a selective NLRP3 inflammasome inhibitor.

The NLRP3 inflammasome is activated through a two-step process involving priming and activation [33]. To determine whether ML345 interferes with priming, BMDMs received pre- or post-LPS treatment with ML345. The expression of NLRP3, pro-IL-1β, pro-caspase-1, ASC, and NEK7, as well as the secretion of IL-6 and TNF, remained unchanged in the presence of ML345 (Fig. 3f–h). ML345 also did not affect LPS-induced phosphorylated or total NF-κB p65 (Fig. 3i). Thus, ML345 does not impact the NF-κB–dependent priming phase, supporting its selective inhibition of NLRP3.

Previous studies have identified several inhibitors of the NLRP3 inflammasome, including MCC950, oridonin, CY-09, tranilast, RRx-001, and entrectinib [21, 22, 34–37]. We compared ML345 with these inhibitors and found that MCC950 remained the most potent, while ML345 showed comparable efficacy to RRx-001 and greater potency than oridonin, CY-09, tranilast, and entrectinib (Fig. S1a–f). However, in a phase II rheumatoid arthritis trial, MCC950 was associated with elevated serum liver enzymes, indicating potential hepatotoxicity [11]. Consistent with previous reports showing that oridonin, tranilast, and RRx-001 inhibit NF-κB signaling [22, 35, 36], we observed that these compounds reduced IL-6 release (Fig. S1g), indicating a general anti-inflammatory response rather than selective inhibition of NLRP3. In contrast, ML345 selectively suppressed NLRP3 inflammasome activation without affecting NF-κB–dependent priming (Fig. 3f–i and Fig. S1g). Taken together, the evidence supports that ML345 functions as a potent and selective inhibitor of the NLRP3 inflammasome.

### ML345 disrupts the NEK7–NLRP3 interaction to prevent inflammasome activation

**Fig. 4:**
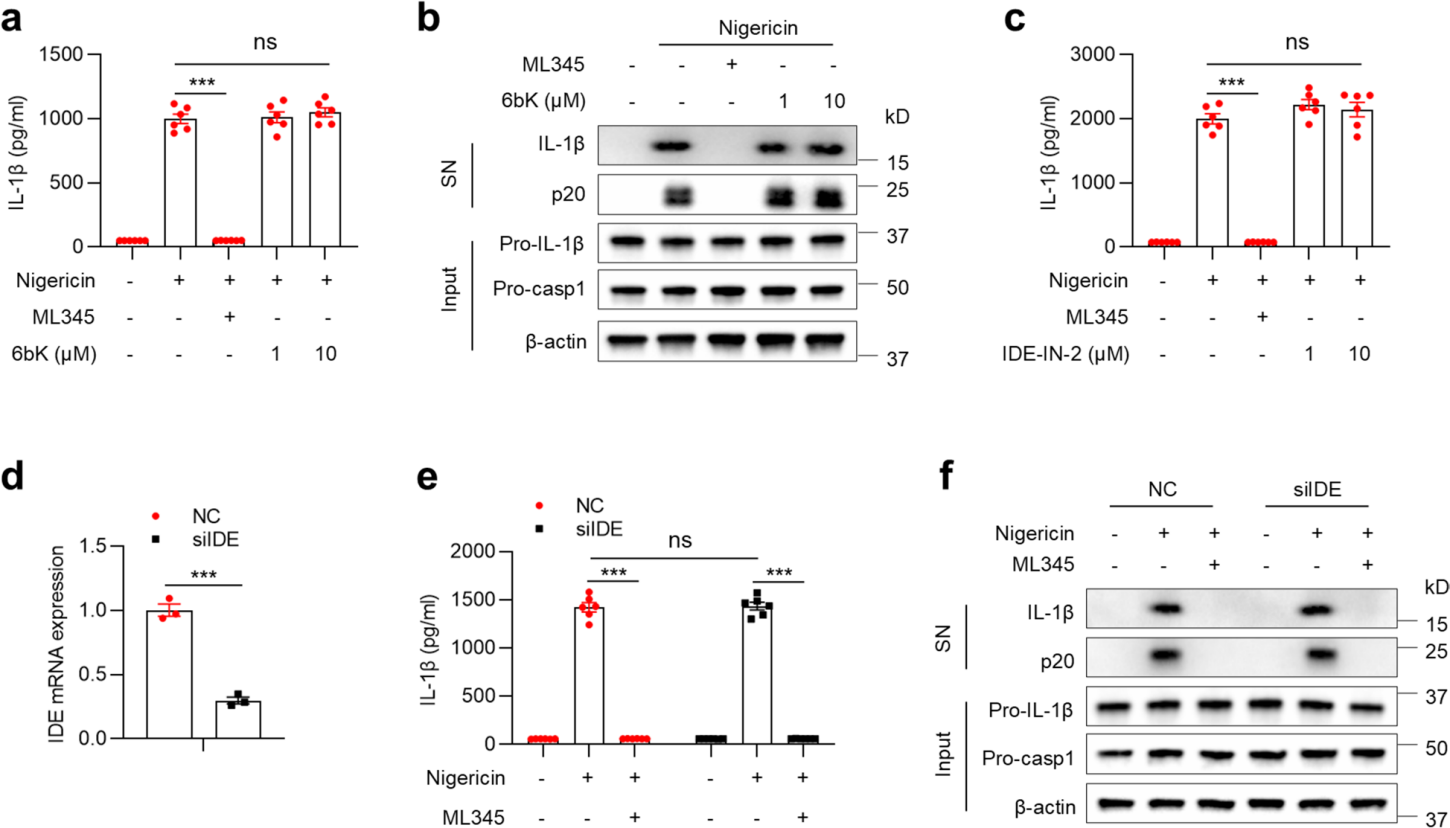
ML345 inhibits NLRP3 inflammasome activation independently of IDE. **a–c** BMDMs were treated with ML345, 6bK, or IDE-IN-2 and then stimulated with nigericin. **a**,** c** IL-1β levels in SN were measured by ELISA. **b** Immunoblot showing secreted IL-1β and p20 in SN, and pro-IL-1β/pro-casp1 in input. **d–f** BMDMs transfected with siRNA were treated with ML345 before nigericin stimulation. **d** IDE expression in BMDMs transfected with siRNA. **e** IL-1β secretion measured by ELISA. (**f**) Immunoblot as described above. Data are mean ± SEM from six (**a**,** c**,** e**) or three (**d**) independent experiments. Statistical significance was assessed using two-way ANOVA (**a**,** c**,** e**) or an unpaired Student’s t-test (**d**). ***p < 0.001, ns, not significant

The mechanism underlying ML345 inhibition of NLRP3 was subsequently investigated. IDE has been reported as a molecular target of ML345 [25]. To test whether ML345 acts via IDE, BMDMs were treated with the selective IDE inhibitors 6bK or IDE-IN-2. However, neither treatment affected IL-1β secretion or caspase-1 cleavage (Fig. 4a–c). Likewise, IDE silencing with siRNA did not alter NLRP3 inflammasome activation or reverse ML345 inhibition (Fig. 4d–f), indicating that the effect of ML345 is independent of IDE.

NLRP3 inflammasome activation is driven by two well-established upstream events: potassium efflux and mitochondrial ROS production [38–41]. Nigericin markedly reduced intracellular potassium levels, but ML345 did not prevent this change (Fig. S2a). ML345 also inhibited IL-1β release induced by CL097, an agonist independent of potassium efflux (Fig. S2b) [28]. MitoSOX staining further showed that ML345 did not alter mitochondrial ROS production (Fig. S2c-d). Thus, ML345 does not affect upstream events of NLRP3 activation.

**Fig. 5:**
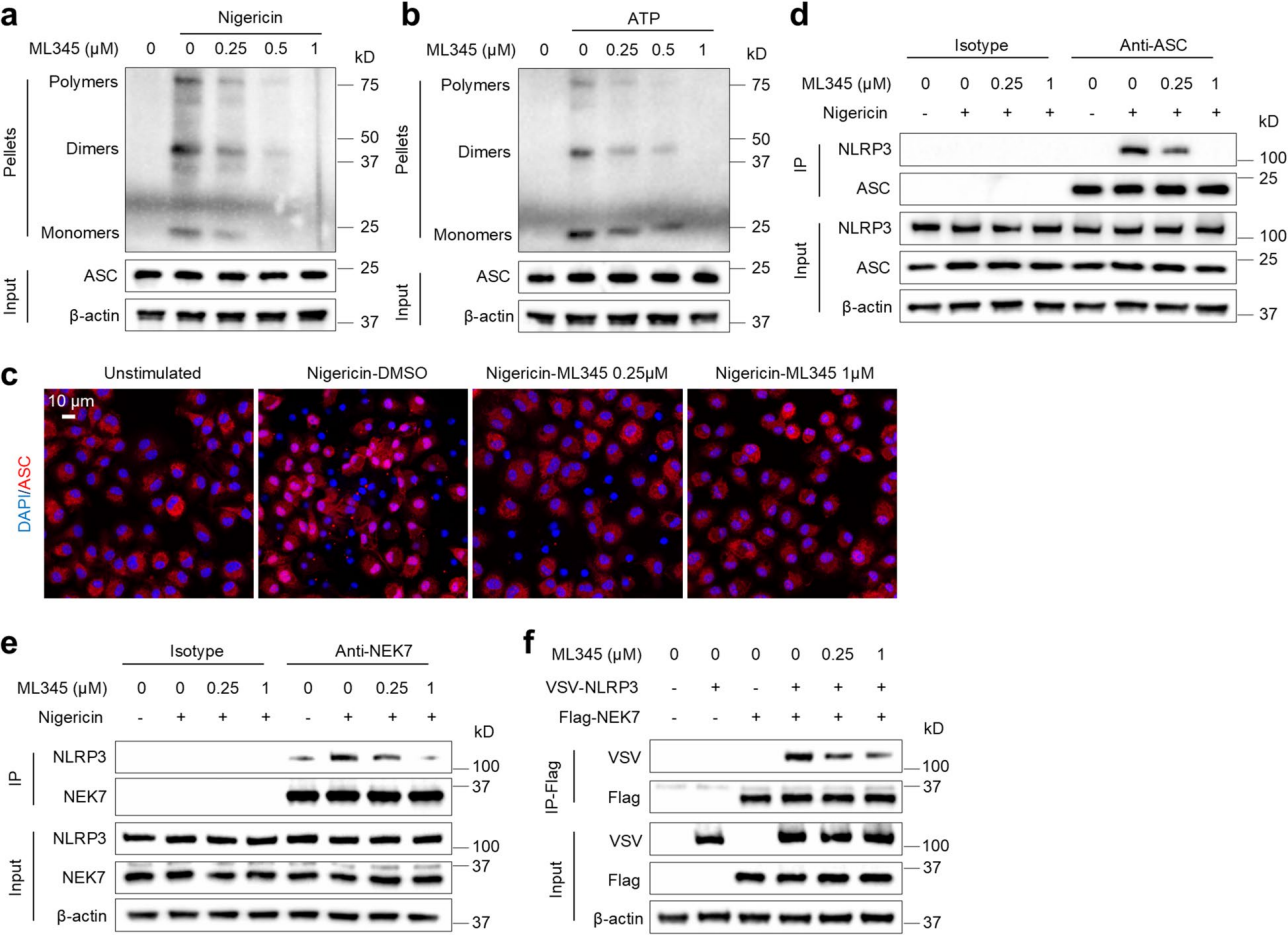
ML345 inhibits NLRP3 inflammasome assembly by disrupting the interaction between NEK7 and NLRP3. **a** and** b** BMDMs were treated with ML345 and then stimulated with nigericin or ATP. ASC oligomerization was analyzed by immunoblot. **c–e** BMDMs were treated with ML345 before nigericin stimulation. **c** Immunofluorescence of ASC specks. **d** IP and immunoblot of endogenous NLRP3–ASC interaction. **e** IP and immunoblot of endogenous NEK7–NLRP3 interaction. **f** IP and immunoblot of exogenous NEK7–NLRP3 interaction

Because ML345 functions independently of IDE and upstream events, we next examined inflammasome assembly. ML345 inhibited ASC oligomerization triggered by nigericin or ATP (Fig. 5a-b) and reduced ASC speck formation (Fig. 5c). Furthermore, ML345 substantially abolished endogenous NLRP3–ASC binding in BMDMs and reduced their colocalization (Fig. 5d, Fig. S3a-b). NEK7 binding to NLRP3 is required for NLRP3 oligomerization and formation of the NLRP3–ASC complex. ML345 significantly disrupted endogenous NEK7–NLRP3 association and reduced their colocalization (Fig. 5e, Fig. S3c-d). These results indicate that ML345 prevents NLRP3 inflammasome formation, likely by directly disrupting NEK7–NLRP3 binding.

Consistent with this hypothesis, ML345 disrupted the direct NEK7–NLRP3 binding (Fig. 5f). By contrast, ML345 had no effect on NLRP3 self-interaction or NLRP3–ASC interaction (Fig. S4a-b). Moreover, unlike the NLRP3 inhibitor CY-09, ML345 did not affect NLRP3 ATPase activity (Fig. S4c). Together, these findings demonstrate that ML345 impedes inflammasome complex formation through direct disruption of NEK7–NLRP3 binding.

### ML345 binds to the Y381 residue of NLRP3 to exert its inhibitory effect

**Fig. 6:**
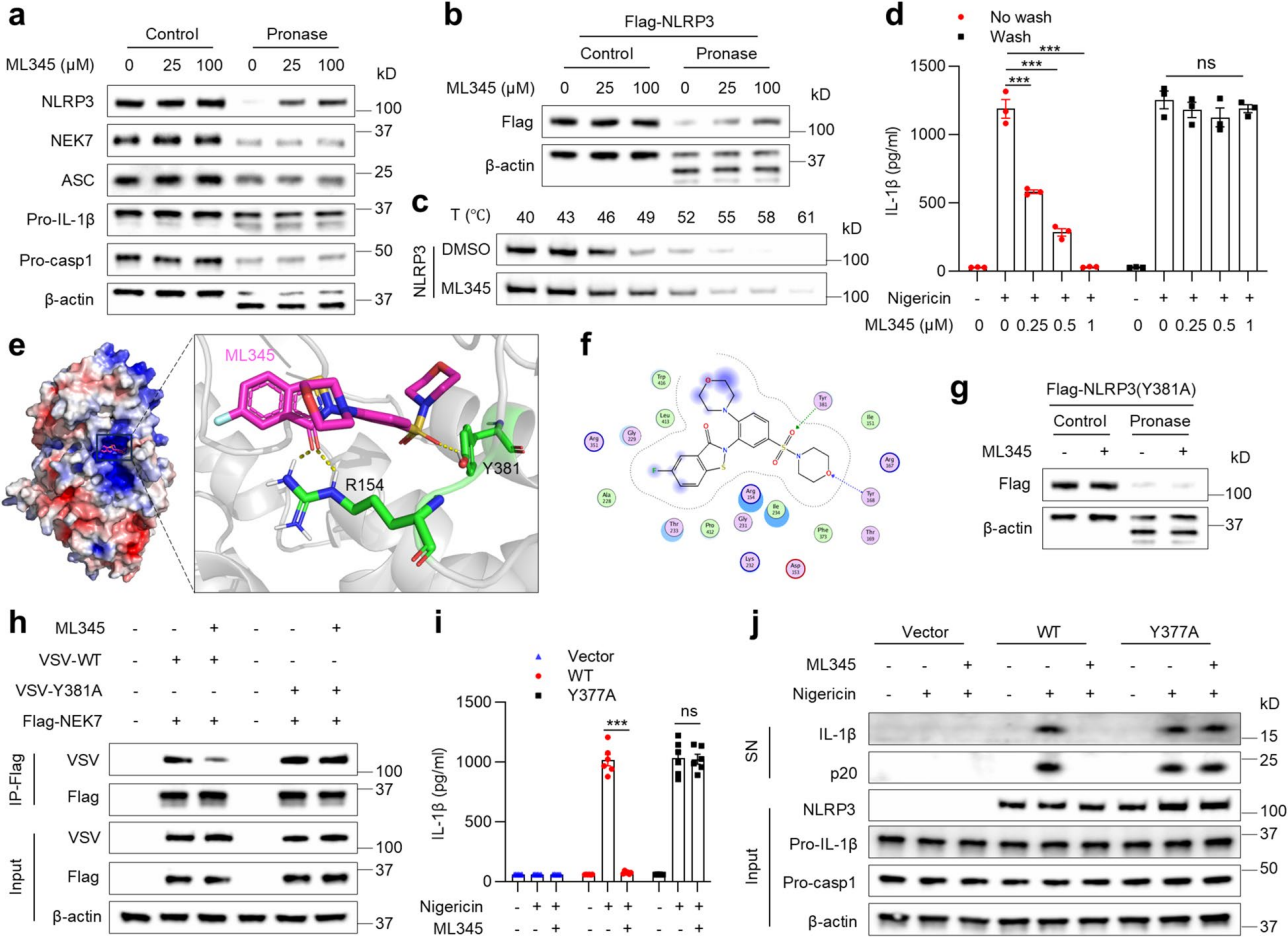
ML345 binds to Y381 of NLRP3. **a** and **b** Protein stability was assessed by DARTS with immunoblotting of (**a**) NLRP3, NEK7, ASC, pro-IL-1β, and pro-casp1 in BMDMs or (**b**)Flag-NLRP3 in HEK-293 T cells, treated with ML345. **c** NLRP3 stability was evaluated by CETSA and immunoblot in BMDMs treated with ML345. **d** IL-1β levels in SN were measured by ELISA. **e** Molecular docking model of the NLRP3–ML345 complex. ML345 depicted as sticks in light red, NLRP3 (PDB: 6NPY) as a gray cartoon, with critical residues highlighted. **f** 2D interaction diagram of ML345 bound to NLRP3. **g** DARTS with immunoblotting of NLRP3 in HEK-293 T cells expressing Y381A Flag-NLRP3. **h** IP and immunoblot of NEK7 interaction with NLRP3 (WT or Y381A). **i** and **j** *Nlrp3*^*⁻/⁻*^ BMDMs expressing WT or Y377A NLRP3 were treated with ML345 before nigericin stimulation. **i** IL-1β secretion was measured by ELISA. (**j**) Immunoblot showing secreted IL-1β and p20 in SN, and pro-IL-1β/pro-casp1 in input. Data are mean ± SEM from three (**d**) or six (**i**) independent experiments. Statistical significance was assessed using one-way ANOVA (**d**) or two-way ANOVA (**i**). ***p < 0.001, ns, not significant

Since ML345 disrupts the direct NEK7–NLRP3 interaction, its direct binding to NEK7 or NLRP3 was tested using a drug affinity responsive target stability (DARTS) assay, which detects target engagement by measuring reduced protease susceptibility upon ligand binding [22, 42]. ML345 dose-dependently protected NLRP3 from pronase-mediated degradation, but did not affect NEK7, ASC, pro-IL-1β, or pro-caspase-1 (Fig. 6a), indicating a direct interaction between ML345 and NLRP3. ML345 similarly conferred dose-dependent protection to Flag-tagged NLRP3, whereas NLRP1b, AIM2, and NLRC4 remained unaffected (Fig. 6b and Fig. S5a–c), confirming its specificity for NLRP3. Consistently, cellular thermal shift assay (CETSA) showed that ML345 markedly increased NLRP3 thermal stability in BMDMs (Fig. 6c). Together, these results establish a direct and selective interaction between ML345 and NLRP3.

To further characterize this interaction, we examined reversibility. LPS-primed BMDMs were pretreated with ML345, washed to remove non-covalently bound compound, and then stimulated with nigericin. After washout, ML345 no longer inhibited IL-1β secretion (Fig. 6d), indicating reversible, non-covalent binding of ML345 to NLRP3.

Next, we aimed to identify the binding site. Molecular docking predicted hydrogen bonds between ML345 and arginine 154 (R154) and tyrosine 381 (Y381) of NLRP3 (binding energy –8.26 kcal/mol; Fig. 6e-f). DARTS assays with NLRP3 mutants revealed that the Y381A, but not R154A, completely abolished ML345 binding (Fig. 6g and Fig. S5d), confirming Y381 as the critical residue.

We then assessed functional relevance. ML345 disrupted NEK7 binding to wild-type NLRP3 but not to the Y381A mutant (Fig. 6h). Similarly, in NLRP3^−/−^ BMDMs reconstituted with WT or Y377A mouse NLRP3 (homologous to human Y381A), ML345 suppressed IL-1β release and caspase-1 cleavage only in WT cells (Fig. 6i-j). Collectively, these results demonstrate that ML345 inhibits NLRP3 inflammasome assembly and activation by non-covalently binding to the Y381 residue of NLRP3.

### ML345 inhibits LPS-induced systemic inflammation and miscarriage

**Fig. 7:**
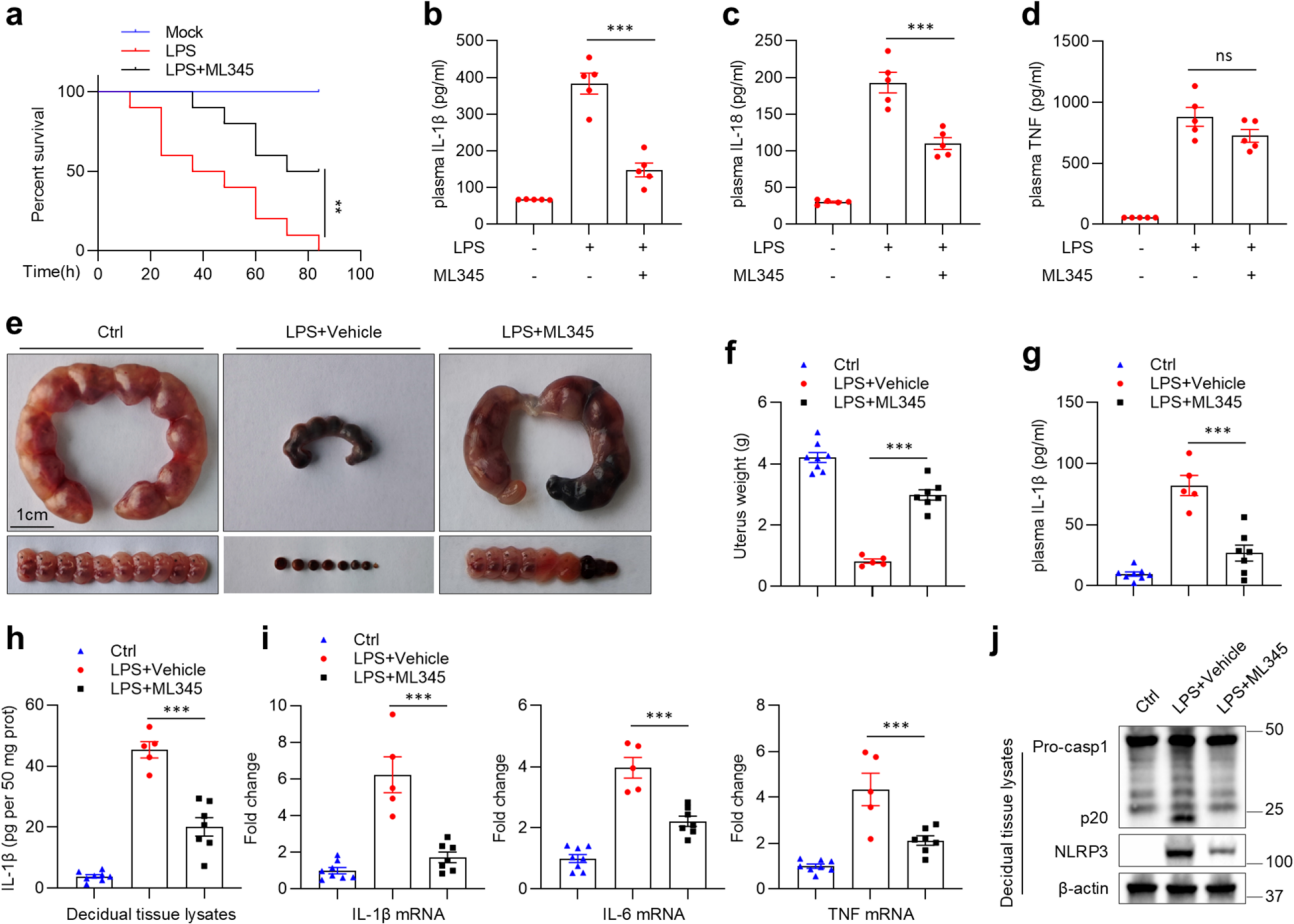
ML345 exerts therapeutic effects in mouse models of systemic inflammation and miscarriage.** a** Survival of normal and LPS-challenged mice treated with vehicle or ML345 (*n* = 10). **b–d** Serum IL-1β (**b**), IL-18 (**c**), and TNF (**d**) in normal and LPS-challenged mice treated with vehicle or ML345 (*n* = 5). **e–j** Pregnant control (*n* = 8) and LPS-challenged mice were treated with vehicle (*n* = 5) or ML345 (*n* = 7): (**e**) representative uterus and fetuses images, (**f**) uterus weight, (**g**) serum IL-1β, (**h**) IL-1β in decidua, (**i**) qRT-PCR of IL-1β, IL-6, and TNF mRNA in decidua, and (**j**) immunoblot of P20 and NLRP3 in decidua. Data are mean ± SEM. Statistical significance was assessed using generalized Wilcoxon test (**a**) or one-way ANOVA (**b–d**, **f–i**). **p < 0.01, ***p < 0.001, ns, not significant

ML345 suppressed NLRP3 activation in vitro, prompting us to explore its anti-NLRP3 efficacy in vivo. LPS-induced systemic inflammation, a widely recognized acute model dependent on NLRP3, triggers robust IL-1β production following intraperitoneal injection [22]. Mice were monitored for survival or sacrificed 4 h later for cytokine analysis. ML345 treatment significantly improved survival and selectively reduced serum IL-1β and IL-18, while minimally affecting NLRP3-independent TNF (Fig. 7a–d). These results reveal that ML345 effectively suppresses NLRP3-driven systemic inflammation in vivo.

Miscarriage, a distressing pregnancy complication often triggered by infection, induces NLRP3 expression and activation, making the inflammasome a potential therapeutic target [43, 44]. On gestational day (Gd) 7.5, pregnant mice received an intraperitoneal injection of LPS (0.25 mg/kg), and ML345 or vehicle was subsequently administered at Gd 7.5, 9.5, and 11.5 (Fig. S6a). At Gd 13.5, LPS-induced fetal resorption was evident, while ML345 significantly reduced the fetal resorption rate and mitigated reductions in uterus weight (Fig. 7e-f and Fig. S6b). In peripheral blood, IL-1β levels were markedly elevated in vehicle-treated mice but significantly attenuated by ML345 (Fig. 7g). In the decidua, ML345 treatment similarly decreased IL-1β protein and the mRNA levels of IL-1β, IL-6, and TNF (Fig. 7h-i). Moreover, ML345 inhibited caspase-1 cleavage and downregulated NLRP3 protein in the decidua, where NLRP3 was predominantly expressed (Fig. 7j and Fig. S6c). Collectively, these findings indicate that ML345 efficiently ameliorates LPS-induced miscarriage by restraining NLRP3 inflammasome activation and associated inflammatory responses.

To evaluate translational potential, naive mice received intraperitoneal ML345 (10 mg/kg) or vehicle every other day for three months. ML345 had no effect on body weight and did not induce observable toxicity in the heart, liver, spleen, lungs, or kidney, nor did it alter fed blood glucose levels or serum levels of ALT, AST, ALP, TBIL, LDH, urea, or creatinine (Fig. S7a–c). Moreover, in healthy pregnant mice, ML345 treatment had no impact on fasting blood glucose levels or glucose tolerance and did not affect uterus weight or fetal weights (Fig. S7d–g). These results indicate that ML345 is both efficacious and well-tolerated in vivo, underscoring its strong potential for clinical translation.

## Discussion

In this study, ML345 was identified as a safe, potent, and selective NLRP3 inhibitor that restrains inflammasome activation and mitigates NLRP3-driven pathologies, including systemic inflammation and miscarriage. These findings indicate that ML345 effectively modulates NLRP3 activation and represents a promising therapeutic candidate.

Aberrant NLRP3 inflammasome activation contributes to diverse inflammatory disorders, including sepsis, CAPS, peritonitis, atherosclerosis, and experimental autoimmune encephalomyelitis (EAE), underscoring its therapeutic relevance. ML345 inhibited IL-1β release with an IC₅₀ of 197.7 nM and fully suppressed NLRP3 inflammasome activation at 1 μM, demonstrating superior potency compared to most reported inhibitors. Importantly, ML345 inhibited NLRP3 activation across both murine and human systems, supporting its translational potential. Intraperitoneal administration of ML345 at 10 mg/kg/day significantly protected mice from systemic inflammation and miscarriage, thereby confirming its efficacy in vivo.

ML345 selectively inhibited canonical, noncanonical, and alternative NLRP3 pathways without affecting other inflammasomes or NF-κB–dependent priming. It directly and reversibly interacts with NLRP3, without targeting other inflammasome components or sensors. Safety was supported by the absence of cytotoxicity in BMDMs and by long-term mouse studies showing no changes in body weight, liver and kidney function, or major organ histology. Together, these results demonstrate that ML345 is a well-tolerated and highly selective NLRP3 inhibitor. Notably, unlike covalent drugs, which may cause idiosyncratic toxicity or immune-mediated hypersensitivity [45], ML345’s non-covalent binding to NLRP3 likely underlies its favorable safety profile.

Although previously identified as a small-molecule inhibitor of IDE, ML345 exerts its NLRP3 inflammasome-inhibitory activity independently of IDE, acting instead by directly targeting NLRP3. ML345 did not affect potassium efflux or mitochondrial ROS production, two critical upstream events in NLRP3 activation. Mechanistically, ML345 binds directly to the conserved Y381 residue of NLRP3, disrupting its interaction with NEK7 and preventing inflammasome assembly. While structural analysis indicates that Y381 is not located at the NEK7–NLRP3 interface [46], ML345 may induce a conformational change in NLRP3 that indirectly disrupts this interaction. Nonetheless, the precise structural basis underlying this effect remains to be clarified.

To assess the pharmacological profile of ML345, we compared its efficacy and selectivity with reported NLRP3 inhibitors, including MCC950, oridonin, CY-09, tranilast, RRx-001, and entrectinib. ML345 exhibited inhibitory activity comparable to RRx-001 and greater potency than oridonin, CY-09, tranilast, and entrectinib. While MCC950 is highly potent, its clinical application is limited by hepatotoxicity observed in a phase II trial, and oridonin, tranilast, and RRx-001 broadly inhibit NF-κB signaling, suggesting off-target anti-inflammatory effects. In contrast, ML345 selectively inhibited NLRP3 without affecting NF-κB-mediated priming or other inflammasome pathways. These findings highlight the superior selectivity and translational promise of ML345 as a next-generation NLRP3 inhibitor.

Our findings demonstrate the therapeutic efficacy of ML345 in mouse models of systemic inflammation and miscarriage. In LPS-exposed mice, ML345 reduced NLRP3 expression and IL-1β levels in serum and decidua, confirming potent in vivo inhibition of NLRP3. In the miscarriage model, ML345 not only decreased IL-1β levels but also reduced IL-6 and TNF, which are independent of NLRP3. This likely reflects inhibition of a secondary immune response, in which IL-1β released from inflammasome activation triggers immune cell infiltration and upregulation of other inflammatory cytokines.

Miscarriage affects approximately 15% of clinically recognized pregnancies, causing considerable emotional distress and often being associated with infection [47]. Although NLRP3 expression is upregulated in miscarriage, its therapeutic relevance has not been fully established. In this study, ML345 ameliorated LPS-induced miscarriage by inhibiting NLRP3 inflammasome activation. NLRP3 expression was predominantly localized to the decidua, where ML345 exerted its protective effects. However, ML345’s effects on NLRP3 in other uterine compartments, such as the placenta and trophoblast, have not yet been characterized and require further investigation. Importantly, ML345 showed no adverse effects on embryonic development in healthy pregnant mice, suggesting that it can be safely used during pregnancy. These results support the potential of NLRP3-targeted therapies for the clinical treatment of recurrent miscarriage.

Interestingly, ML345 did not induce hypoglycemia or impair glucose tolerance under our experimental conditions, despite its reported IDE inhibitory activity. This discrepancy may be attributed to the relatively low dosing regimen and limited treatment duration, as well as possible differences in the animal model used.

Although this work provides valuable insights, certain limitations should be noted. First, based on molecular docking predictions, Y381 was identified as a key residue mediating ML345’s inhibition of NLRP3. Nevertheless, the potential involvement of additional residues cannot be excluded, and further structural studies are needed to fully elucidate its mechanism. Second, ML345’s therapeutic potential in type 2 diabetes (T2D) remains to be determined. Future studies in T2D models, including those using IDE-deficient mice, are necessary to distinguish the relative contributions of IDE inhibition and NLRP3 suppression and to evaluate the potential of ML345 as a dual-targeting therapy.

Current clinical strategies for NLRP3-related diseases primarily rely on IL-1β-targeting biologics. Direct inhibition of NLRP3 using highly specific small molecules such as ML345 may provide distinct therapeutic advantages. Although several NLRP3 inhibitors have recently been identified, their clinical efficacy and safety remain unconfirmed, and to date, no therapy targeting NLRP3 has received regulatory approval. In this study, ML345 exhibited potent, selective, and well-tolerated anti-inflammasome activity in cellular and animal models. These findings support ML345 as a promising lead candidate targeting NLRP3-related pathologies.

## Materials and methods

### Reagents

ML345 (HY-117878), imiquimod (HY-B0180), Tranilast (HY-B0195), MCC950 (HY-12815), 6bK (HY-110197), IDE-IN-2 (HY-W157689), CY-09 (HY-103666), and nigericin (HY-127019) were purchased from MedChemExpress. Oridonin (S2335) and RRx-001 (S8405) were obtained from Selleck. Entrectinib (T3678) was purchased from TargetMol. Lipopolysaccharide (LPS, tlrl-peklps), Lipofectamine 2000 (11,668,019), Pam3CSK4 (tlrl-pms), MitoSOX (M36008), DAPI (D21490), and SiO₂ (tlrl-sio-2) were purchased from Invitrogen. Alum (77,161) was acquired from Thermo Fisher Scientific, and M-CSF (CB34) was from Novoprotein. ATP (34,369–07–8), MSU (69–93-2), pronase (P5147), Protein G agarose (P7700), poly(dA:dT) (86,828–69-5), phorbol 12-myristate 13-acetate (PMA, P8139), as well as anti-Flag beads (A2220) were obtained from Sigma-Aldrich.

### Mice

C57BL/6 J mice were obtained from Beijing Huafukang Biotechnology Co., Ltd. (Beijing, China). *Nlrp3⁻*^*/*^*⁻* mice were generated as previously reported [48]. All mice were maintained under specific pathogen-free conditions with ad libitum access to food and water, at 24–26 °C and 65–70% humidity, under a 12-h light/dark cycle.

### Cell lines

Primary BMDMs from C57BL/6 mice were cultured for 4–6 days in DMEM containing 10% FBS, 1% penicillin–streptomycin (P/S), and 20 ng/mL murine M-CSF. Human PBMCs were obtained from fresh peripheral blood and maintained in RPMI 1640 containing 10% FBS and 1% P/S. HEK-293 T and iBMDM cells were maintained in DMEM with 10% FBS and 1% P/S, while THP-1 cells were cultured in RPMI 1640 supplemented with the same components. All cell lines were used at passages 5**–**15.

### Inflammasome stimulation and activation assays

BMDMs were primed with LPS (50 ng/mL, 3 h), then pretreated with inhibitors (30 min), and stimulated with various activators: nigericin (5 μM, 30 min), ATP (2.5 mM, 30 min), MSU (150 μg/mL, 4 h), poly(dA:dT) (0.5 μg/mL, 4 h, transfection), SiO₂ (300 μg/mL, 6 h), alum (300 μg/mL, 6 h), imiquimod (15 μg/mL, 3 h), TcdB (0.5 μg/mL, 1 h), or infection with Salmonella (MOI 5) or Listeria (MOI 10) for 1 h followed by gentamicin treatment (3 h). For noncanonical NLRP3 activation, BMDMs were primed with Pam3CSK4 (400 ng/mL, 3 h), pretreated with inhibitors (30 min), and transfected with LPS (0.5 μg/mL, 16 h). PBMCs were primed with LPS (50 ng/mL, 3 h), or pretreated with inhibitors (30 min) before stimulation with nigericin (5 μM, 30 min) or LPS (1 μg/mL, 24 h) for alternative NLRP3 activation. THP-1 cells were differentiated with PMA (100 nM, 4 h), seeded overnight, primed with LPS (200 ng/mL, 3 h), pretreated with inhibitors (30 min), and stimulated with nigericin (5 μM, 1 h) or VbP (10 μM, 24 h). iBMDMs were primed with LPS (50 ng/mL, 3 h), pretreated with inhibitors (30 min), and stimulated with nigericin (5 μM, 2 h). Supernatants were collected for ELISA (R&D Systems) or immunoblotting, lysates were analyzed by immunoblot, and LDH release was measured using a commercial kit (Beyotime).

### Western blotting

Protein samples were boiled, separated by 8–20% SDS–PAGE, and transferred to PVDF membranes. Membranes were blocked with 5% nonfat milk in TBST, incubated with primary and HRP-conjugated secondary antibodies, and visualized using enhanced chemiluminescence. The primary antibodies included anti-Flag (F2555) and anti-VSV (V4888, Sigma-Aldrich), anti-IL-1β (AF-401-NA, R&D Systems), anti-NLRP3 (AG-20B-0014) and anti-caspase-1 (AG-20B-0042, AdipoGen), anti-β-actin (66,009–1-Ig, Proteintech), anti-NEK7 (ab133514, Abcam), and anti-ASC (67824S, Cell Signaling Technology).

### ASC oligomerization assay

BMDMs (1 × 10⁶ cells/well) were stimulated to activate the NLRP3 inflammasome, washed, and lysed in NP-40 buffer. After centrifugation at 330 × g for 10 min at 4 °C, pellets were thoroughly washed with ice-cold PBS, resuspended in 500 μL PBS containing 2 mM DSS (Thermo Fisher Scientific, A39267), and incubated with rotation for 30 min at room temperature prior to immunoblot analysis.

### siRNA interference

BMDMs (3 × 10^5^ cells/well) were transfected in Opti-MEM with 50 nM siIDE using Lipofectamine RNAiMAX (Invitrogen). The siIDE sequence was GAGUAAGCUGUGGUUCAAA.

### Quantitative real-time PCR

Total RNA was extracted with TRIzol, and 1 μg of RNA was reverse transcribed using M-MLV Reverse Transcriptase. Quantitative PCR was performed using SYBR Green premix, and gene expression levels were normalized to GAPDH. Primer sequences for IL-1β, IL-6, TNF, and GAPDH were designed as previously reported [22], and primer sequences for IDE used in this study were as follows:

- IDE forward, TGCAACACCATACCCTGCTC;
- IDE reverse, GTCCACATAAGCAAACGGGC.

### Immunoprecipitation (IP)

To assess endogenous protein interactions, BMDMs (1 × 10⁶ cells/well) were stimulated to activate the NLRP3 inflammasome, washed, and lysed in NP-40 buffer with protease inhibitors. Lysates were incubated overnight at 4 °C with anti-NEK7 or anti-ASC antibodies and Protein G beads under rotation. Immunocomplexes were collected and analyzed by immunoblotting.

To assess exogenous protein interactions, HEK-293 T cells were transfected with plasmids using polyethylenimine (PEI). After 24 h, cells were lysed in NP-40 buffer containing protease inhibitors. Lysates were incubated overnight at 4 °C with anti-Flag antibody-conjugated beads. Immunocomplexes were collected and analyzed by immunoblotting.

### Immunofluorescence staining

BMDMs (2 × 10^5^ cells/well) seeded on coverslips were stimulated to activate the NLRP3 inflammasome, then fixed, permeabilized (4% paraformaldehyde, 0.1% Triton X-100), and blocked. After overnight incubation at 4 °C with primary antibodies (ASC, NLRP3, or NEK7; Santa Cruz), samples were treated with fluorophore-conjugated secondary antibodies. Following DAPI counterstaining, fluorescence images were captured using a Leica confocal microscope.

### Drug affinity responsive target stability assay

Cells were lysed in NP-40 buffer containing protease inhibitors. Protein concentrations were determined using the Pierce BCA Protein Assay Kit (Beyotime). Equal amounts of protein (8 μg per reaction) were incubated with ML345 at 4 °C overnight with rotation, followed by treatment with pronase (25 ng/μg protein) for 30 min at room temperature. Samples were analyzed by immunoblotting.

### Cellular thermal shift assay

LPS-primed BMDMs were treated with ML345 for 2 h, washed three times, and resuspended in PBS. Aliquots (50 μL; 1 × 10⁶ cells per condition) were transferred to PCR tubes and heated at 40–61 °C in 3 °C increments for 5 min, immediately cooled on ice, lysed by three freeze–thaw cycles, and the supernatants were subjected to immunoblotting.

### NLRP3 Reconstitution

*Nlrp3 *^*−/−*^ BMDMs received lentiviral delivery of wild-type or Y377A mouse NLRP3. After 6 h, the medium was replaced with DMEM supplemented with 20 ng/mL murine M-CSF. After 48 h, cells were stimulated to activate the NLRP3 inflammasome.

### Systemic inflammation

Male C57BL/6 mice (8 weeks old) received intraperitoneal injections of ML345 (10 mg/kg) or vehicle. After 30 min, systemic inflammation was induced by intraperitoneal injection of LPS (20 mg/kg). Serum was collected 4 h after LPS administration, and cytokine levels were measured.

### Miscarriage

Male and female C57BL/6 mice (8 weeks old) were housed for natural mating, with vaginal plug detection considered gestational day (GD) 0.5. Pregnant females received LPS (0.25 mg/kg, i.p.) at GD 7.5 to induce miscarriage, followed by ML345 (10 mg/kg) or vehicle at GD 7.5, 9.5, and 11.5. Pregnant mice were sacrificed at designated time points to assess embryo resorption and related parameters.

### Statistical analyses

All results are shown as mean ± SEM. Data were analyzed using an unpaired Student’s t-test for two-group comparisons, and one-way or two-way ANOVA for experiments with multiple groups or two independent variables. No data were omitted from the analysis. Statistical significance was defined as *p* < 0.05.

## Supplementary Information

Supplementary Material 1.

## Data Availability

The research data generated or analyzed during this study are included in this published article and its Supplementary Material. Other data from the corresponding authors are available upon reasonable request.
