# Supplementary material for: ML345 is a potent and selective NLRP3 inflammasome inhibitor with anti-inflammatory activity
Source: Mol Biomed. 2025 Nov 13;6:108. doi: 10.1186/s43556-025-00363-7 (PMC12615901; doi:10.1186/s43556-025-00363-7)
Supplement: Supplementary file 1 — Supplementary Material 1. [file 43556_2025_363_MOESM1_ESM.docx]

**ML345 is a potent and selective NLRP3 inflammasome inhibitor with anti-inflammatory activity**

**Hualong Lin^1^, Xinxin Liang^1^, Weijie Hao^1^, Xiaoli Lu^2,*^, Bo Li^1,*^, Xiaohong Wang^1,*^**

^1^Department of Gynecology and Obstetrics, Tangdu Hospital, Fourth Military Medical University, Xi'an, Shaanxi Province, China

^2^Xi'an International University, 18 Yudou Road, Yanta District, Xi'an, Shaanxi 710077, P.R. China

*** Corresponding author:**

Dr. Xiaohong Wang E-mail: wangxh919@fmmu.edu.cn

Dr. Bo Li E-mail: lbtn2000@126.com

Dr. Xiaoli Lu E-mail: luxiaoli@westlake.edu.cn

**Supplementary Content**

Supplementary figures and figure legends

Supplementary materials and methods


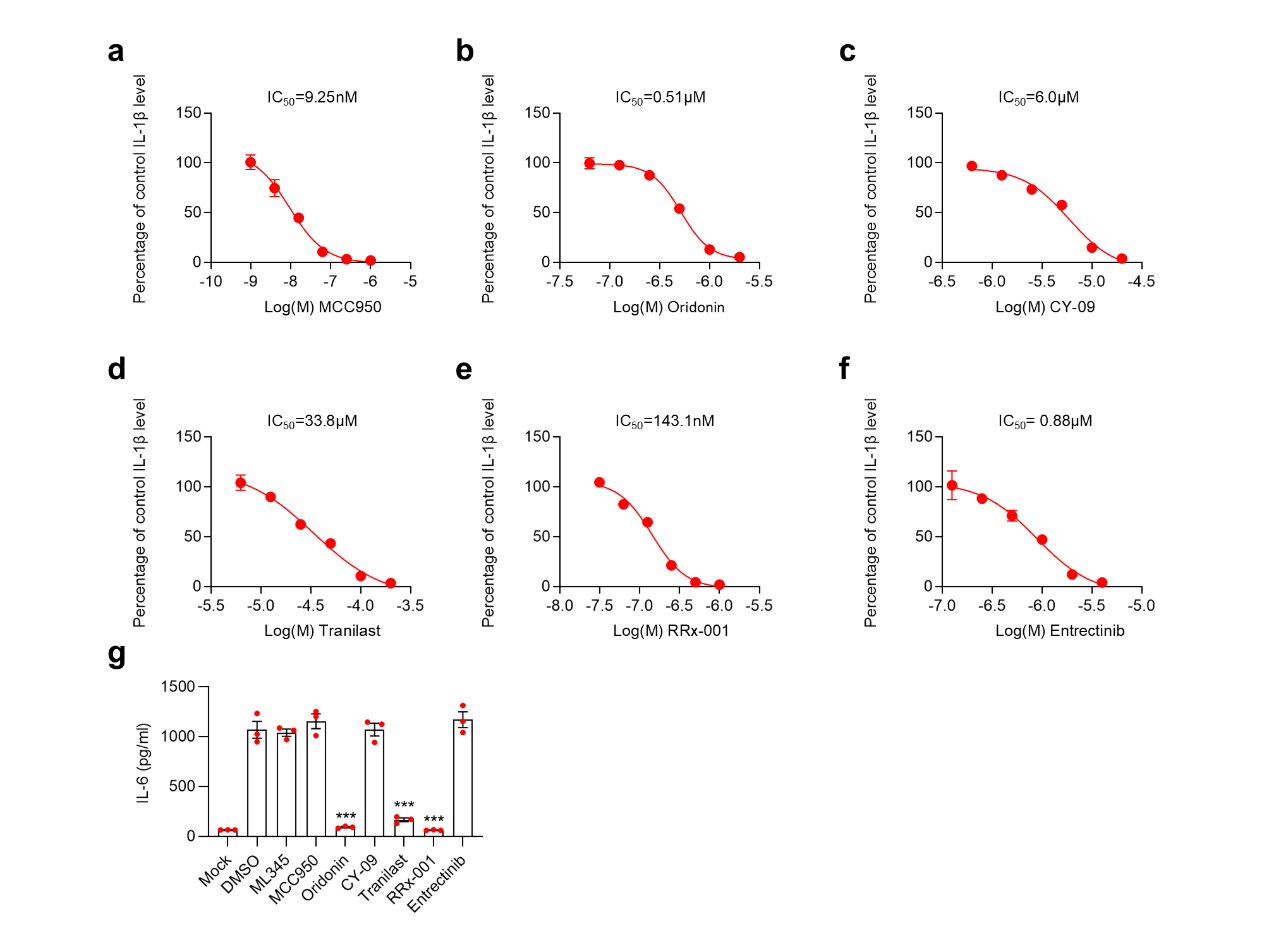
**Supplementary figures and figure legends**

**Fig. S1, related to Fig. 3** Effects of NLRP3 inhibitors on inflammasome activation and NF-κB signaling. **a–f** BMDMs were treated with MCC950(**a**), Oridonin (**b**), CY-09 (**c**), Tranilast (**d**), RRx-001 (**e**) or Entrectinib (**f**) before nigericin stimulation. IL-1β levels in SN were measured by ELISA. **g** BMDMs were treated with inhibitors before LPS stimulation. IL-6 secretion was measured by ELISA. Data are mean ± SEM from three independent experiments. Statistical significance was assessed using one-way ANOVA. ***p < 0.001.


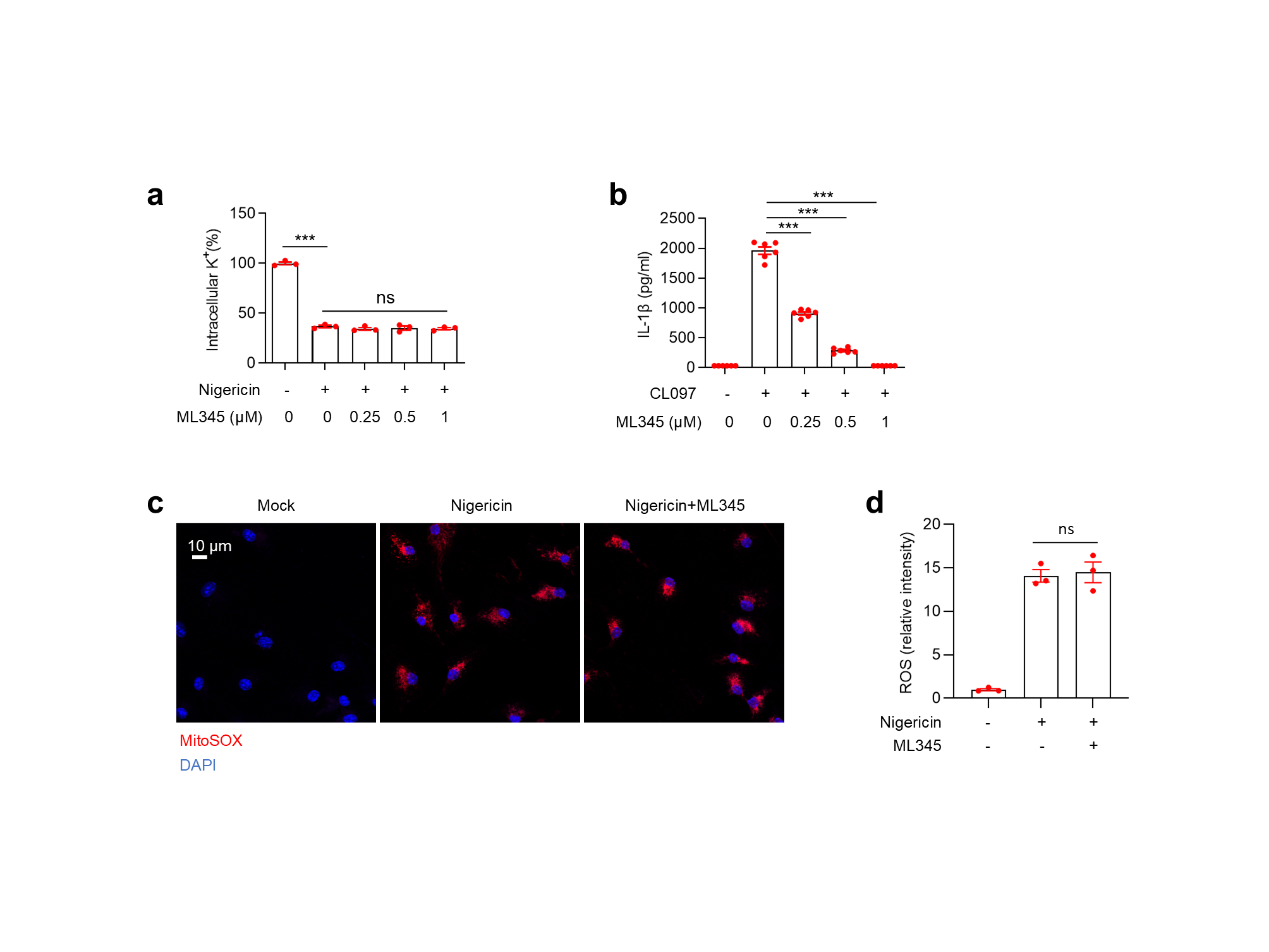
**Fig. S2, related to Fig. 5** ML345 does not affect potassium efflux or mitochondrial ROS production. **a** Intracellular potassium levels in BMDMs treated with ML345 before nigericin stimulation. **b** BMDMs were treated with ML345 before CL097 stimulation. IL-1β levels in SN were measured by ELISA. **c** Confocal microscopy of BMDMs treated with ML345 before nigericin stimulation, then stained with DAPI and MitoSOX. **d** Relative MitoSOX fluorescence. Data are mean ± SEM from three (**a, d**) or six (**b**) independent experiments. Statistical significance was assessed using one-way ANOVA. ***p < 0.001, ns, not significant.


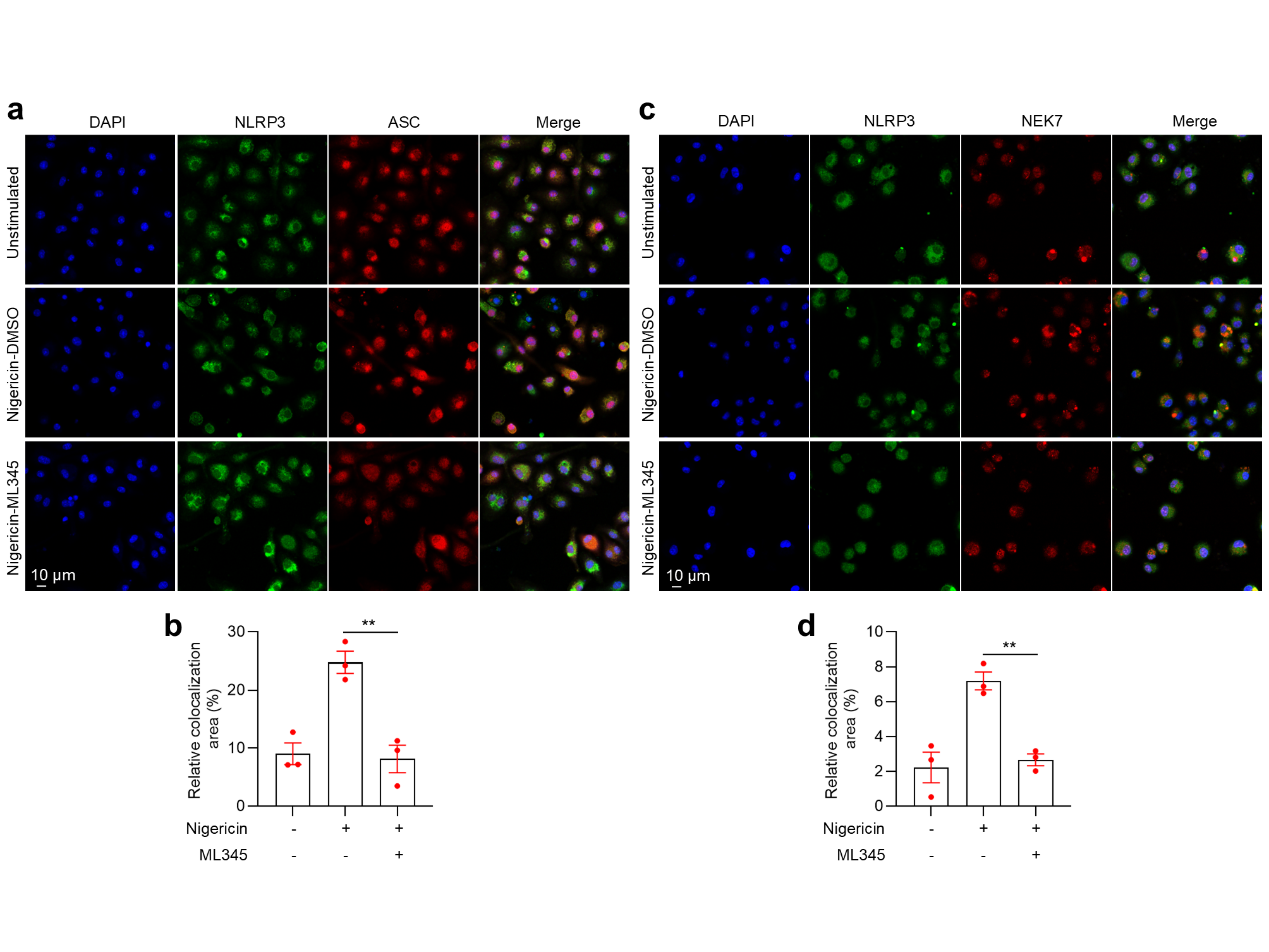
**Fig. S3, related to Fig. 5** ML345 disrupts NLRP3 colocalization with ASC and NEK7. **a** Immunofluorescence of endogenous NLRP3–ASC colocalization. **b** Quantification of NLRP3–ASC colocalized area relative to NLRP3. **c** Immunofluorescence of endogenous NLRP3–NEK7 colocalization. **d** Quantification of NLRP3–NEK7 colocalized area relative to NLRP3. Data are mean ± SEM from three independent experiments. Statistical significance was assessed using one-way ANOVA. **p < 0.01.


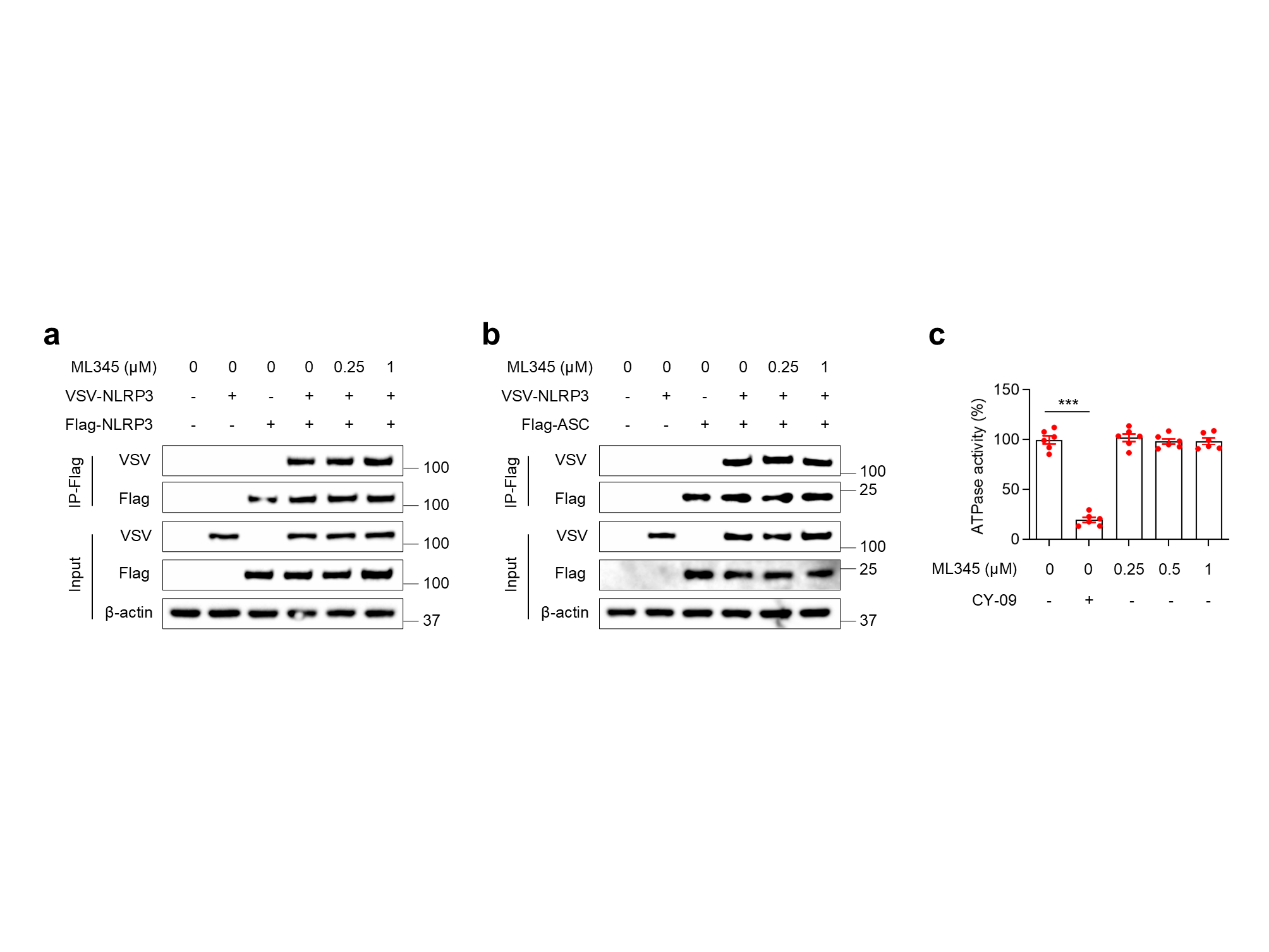
**Fig. S4, related to Fig. 5** ML345 does not affect NLRP3 self-association, its interaction with ASC, or its ATPase activity. **a** IP and immunoblot of exogenous NLRP3–NLRP3 interaction. **b** IP and immunoblot of exogenous NLRP3–ASC interaction. **c** ATPase activity of purified NLRP3 with ML345 or CY-09. Data are mean ± SEM from six independent experiments. Statistical significance was assessed using one-way ANOVA. ***p < 0.001.


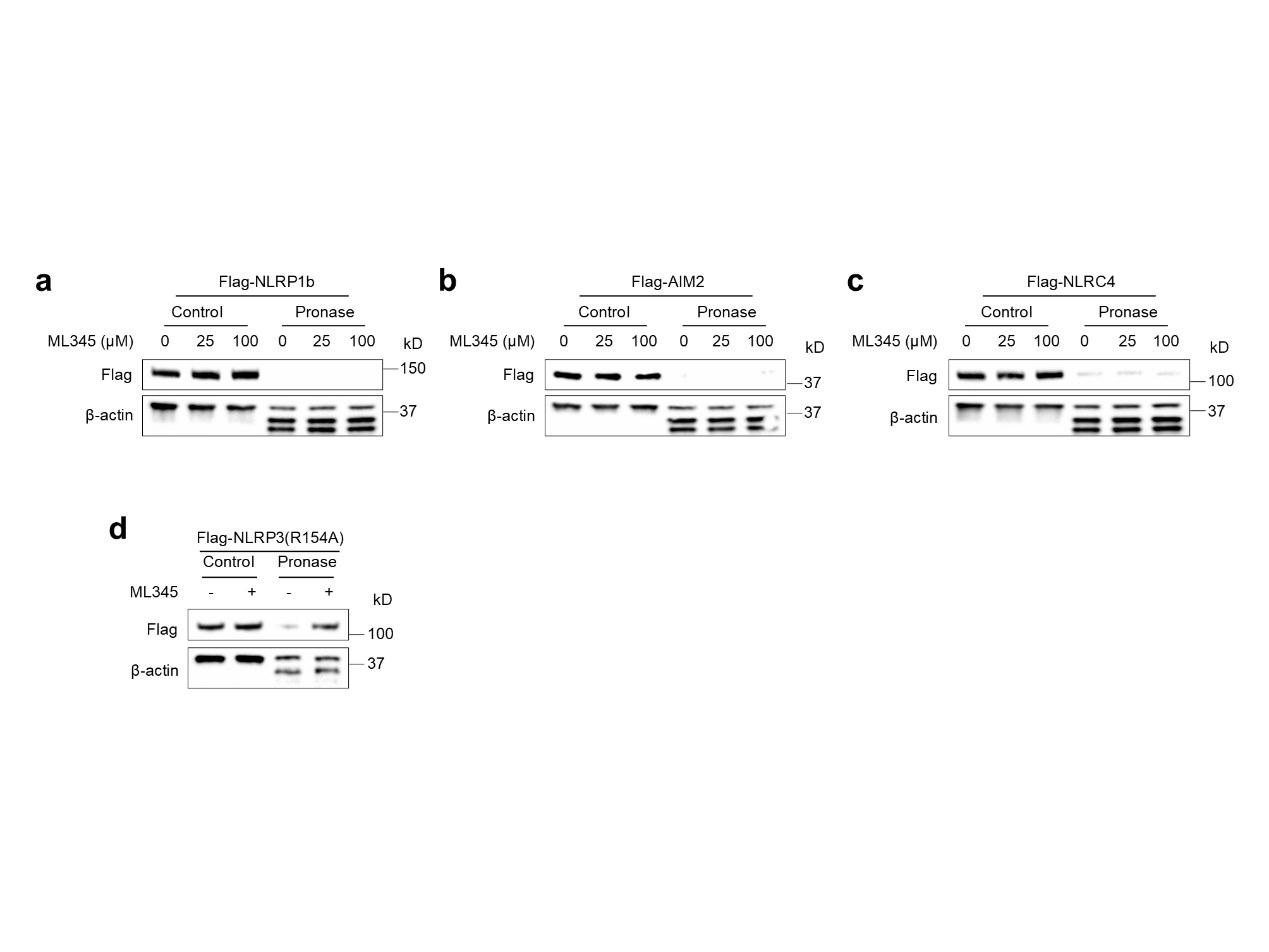
**Fig. S5, related to Fig. 6** ML345 does not affect pronase-mediated degradation of Flag-tagged NLRP1b, AIM2, NLRC4, or the NLRP3 R154A mutant. **a–d** Protein stability was assessed by DARTS with immunoblotting of NLRP1b (**a**), AIM2 (**b**), NLRC4 (**c**), and NLRP3 (R154A) (**d**) in HEK-293T cells, treated with ML345.


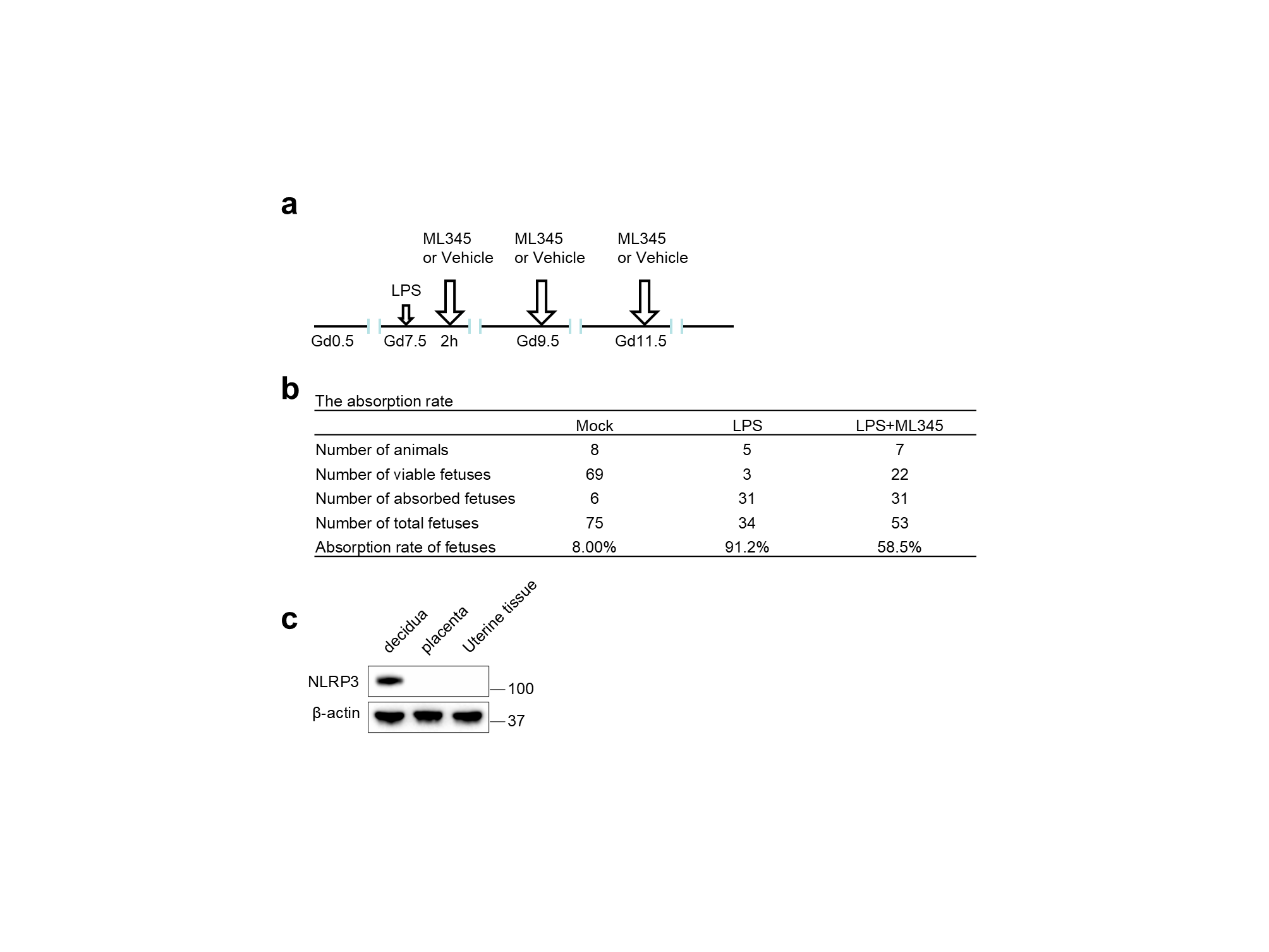
**Fig. S6, related to Fig. 7** ML345 significantly reduced the fetal resorption rate. **a** Schema of ML345 treatment in miscarriage mode. **b** Embryo resorption rate in pregnant control and LPS-challenged mice treated with vehicle or ML345. **c** NLRP3 expression in the decidua, placenta and other uterine tissues of miscarriage mice.


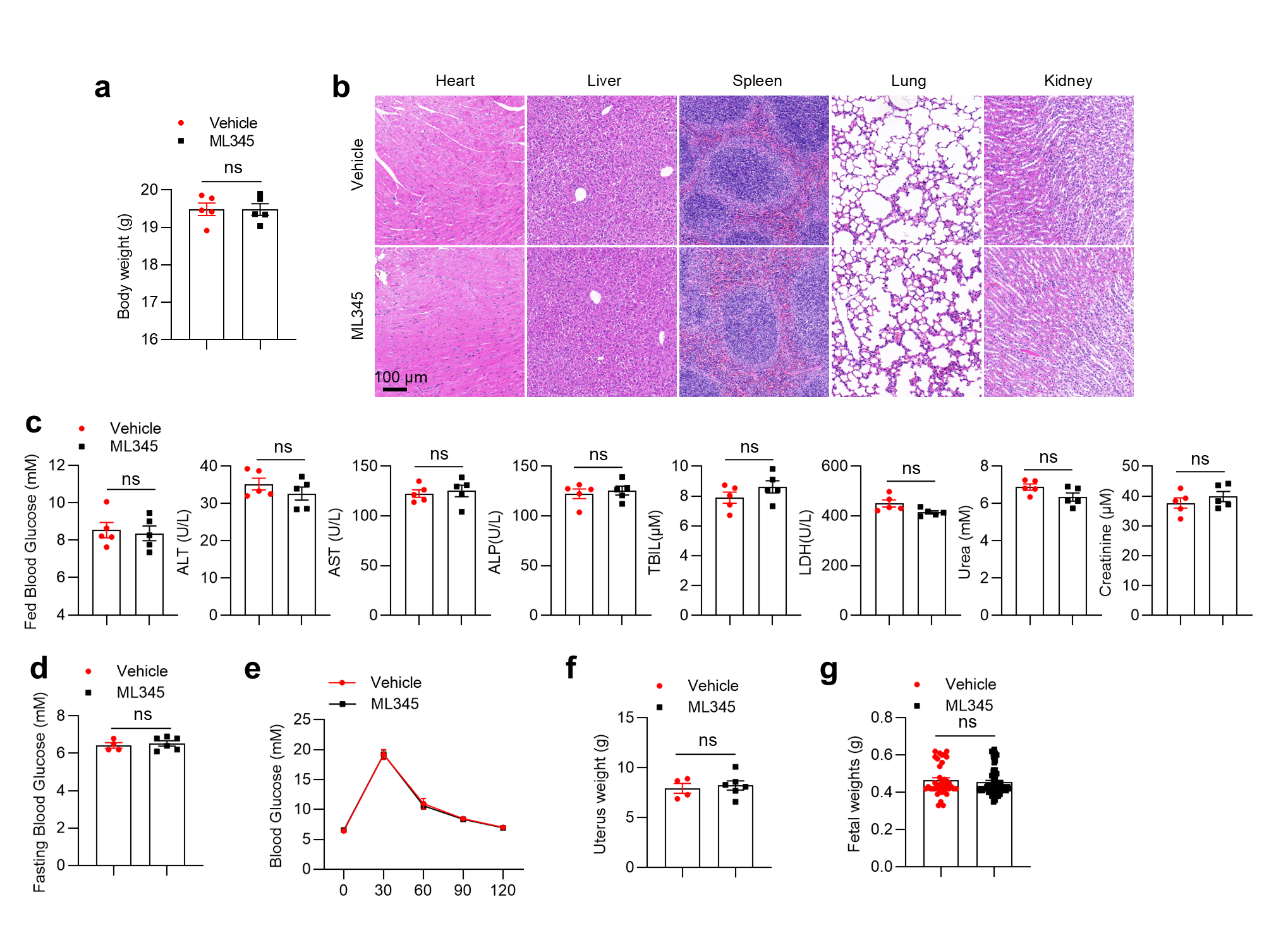
**Fig. S7, related to Fig. 7** ML345 demonstrates good in vivo tolerability. **a–c** C57BL/6J mice were treated with vehicle or ML345 (10 mg/kg) every other day for 3 months (n = 5). (**a**) Body weight. (**b**) H&E staining of heart, liver, spleen, lung, and kidney. (**c**) Fed blood glucose and serum levels of ALT, AST, ALP, TBIL, LDH, urea, and creatinine. **d–g** Pregnant mice were treated with vehicle (n = 4) or ML345 (n = 6) following the dosing regimen described in Fig. S6a. (**d**) Fasting blood glucose. (**e**) Glucose tolerance test. (**f, g**) Uterus weight and fetal weights. Data are mean ± SEM. Statistical significance was assessed using an unpaired Student’s t-test. ns, not significant.

**Supplementary materials and methods**

Intracellular potassium Measurement

BMDMs (1 × 10⁶ cells/well) were stimulated to activate the NLRP3 inflammasome. After stimulation, cells were lysed in 3% ultrapure HNO₃. Lysates were boiled to dryness, resuspended in ddH₂O, and K⁺ concentrations measured using a PerkinElmer Optima 2000 DV spectrometer.

Confocal microscopy

BMDMs (2 × 10⁵ cells/well) were stimulated to activate the NLRP3 inflammasome and incubated with MitoSOX (5 μM, 30 min). Cells were washed three times with ice-cold PBS, fixed with 4% paraformaldehyde for 15 min, and washed with PBST. Nuclei were counterstained with DAPI. Confocal images were acquired using a Leica STELLARIS 5 confocal microscope.

Histological analysis

Fresh mouse tissues were fixed in 4% formalin at 4°C for 24 h, embedded in paraffin, and sectioned. Sections were stained with hematoxylin and eosin (H&E), and images were acquired by light microscopy.

Glucose tolerance test

Pregnant mice were fasted for 6 h and then intraperitoneally injected with 20% (w/v) glucose at 2.0 g/kg body weight. Blood glucose levels were measured at indicated time points from tail vein blood using a glucometer (ACCU-CHEK, Roche).
